# Synchronous Bilateral Testicular Tumors with Different Histopathology

**DOI:** 10.1155/2015/492183

**Published:** 2015-04-28

**Authors:** Ioannis Anastasiou, Dimitrios Deligiannis, Ioannis Katafigiotis, Ioannis Skarmoutsos, Georgios Karaolanis, Viktoria-Varvara Palla, Afrodite Nonni, Dionysios Mitropoulos, Constantinos A. Constantinides

**Affiliations:** ^1^1st University Urology Clinic, Laiko Hospital, University of Athens, 17 Agiou Thoma Street, Attiki, 11527 Athens, Greece; ^2^2nd Department of Surgery, Laiko General Hospital, Medical School of Athens, 17 Agiou Thoma Street, Attiki, 11527 Athens, Greece; ^3^Department of Obstetrics and Gynecology, G. Gennimatas General Hospital, Mesogeion Avenue 154, Attiki, 11527 Athens, Greece; ^4^Department of Pathology, National and Kapodistrian University of Athens, 17 Agiou Thoma Street, Attiki, 11527 Athens, Greece

## Abstract

A 40-year-old male presented to our outpatient department with the chief complaint of a painless mass on his right testis with gradual size increase over the past two months. Physical examination and ultrasound revealed a firm and nontender mass both on the right and on the left testis. The only elevated biomarker was b-hcG (24,7 mIU/mL) and computer tomography (CT) did not reveal any pathology. Bilateral high orchiectomies were performed, without previous frozen storage of the sperm. Histology proved typical seminoma of the left testis and embryonal carcinoma of the right testis. He received two cycles of adjuvant combination chemotherapy with bleomycin, etoposide, and cisplatin. Six months after the operation no residual tumor or recurrence was observed.

## 1. Introduction

Testicular cancer represents between 1% and 1.5% of male neoplasms and 5% of urological tumors in general, with 3–10 new cases occurring per 100,000 males/per year in Western society [1–3]. Only 1-2% of cases are bilateral at diagnosis. Primary bilateral tumors of the testis may occur metachronously in 80–85% of the cases or synchronously in 15–20% of cases [4], but tend to be of the same histologic type. We report a case of a young male presented with synchronous bilateral germ cell tumors (BGCT) with different histology; he underwent bilateral orchiectomy followed by adjuvant chemotherapy. The treatment of patients with synchronous BGCT is based upon the clinical stage and the histopathological type of the tumors and should not be different from the standard management of unilateral testicular carcinoma [4].

## 2. Case Report

**Figure 1:**
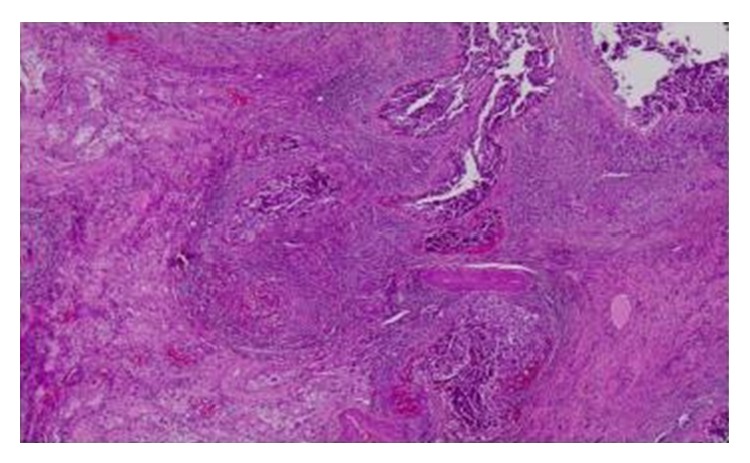
Embryonal cell carcinoma of the right testis.

**Figure 2:**
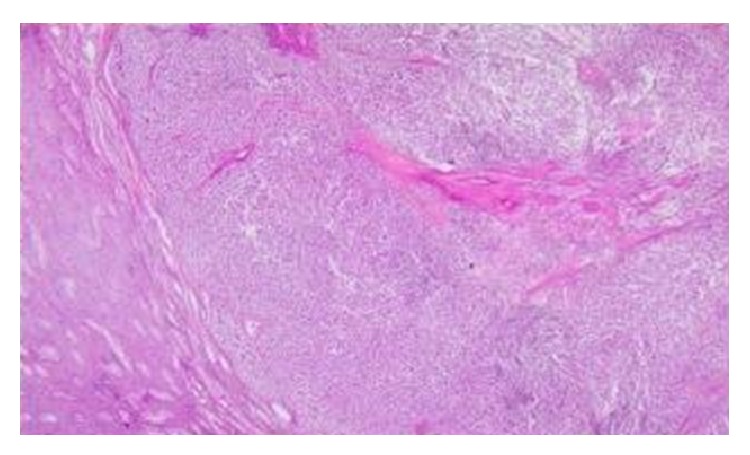
Seminoma of the left testis.

A 40-year-old male presented at our outpatients department complaining about a testicular painless mass on the right testis, which had shown a gradual enlargement over the past two months. The only symptom the patient had was discomfort in the scrotum and a sensation of testicular heaviness. His medical history did not report any known risk factors for testis cancer, such as cryptorchidism, and he did not have any comorbidities. Physical examination revealed a firm and nontender mass both on the right and on the left testis, which were easily separable from the epididymis. No other constitutional signs were present. Laboratory workup revealed a moderately elevated b-hcG (24,7 mIU/mL) and CEA (1,97 ng/mL), a-FP (1,62 ng/mL), and LDH (180 IU/L) within normal levels. Firstly, he was submitted to a scrotal ultrasonography which revealed a testicular mass that measured approximately 2,3 × 3,1 cm on the right testis (Figure 1) and another testicular mass of 2,4 × 1,5 cm in size on the left testis (Figure 2). The computer tomography (CT) of the abdomen did not demonstrate any enlarged retroperitoneal lymph nodes. The chest X-ray showed no abnormality. The patient was scheduled for operation and he did not want to have frozen storage of sperm, although he was fully informed about the consequences, since he was a father of two children and also was informed about the occurrence of hypogonadism after the operation and that he will have to be under a strict endocrinologist follow-up and hormone replacement. He chose bilateral orchiectomy for oncological reasons. Finally, he underwent bilateral orchiectomy with high ligation of the spermatic cord. The postoperative period was uneventful and the patient exited the hospital the next day. The elevated b-hcG on the postoperative measurement (the 15th day) was within normal values (0,3 mIU/mL). Histopathological evaluation of the specimens revealed the following: (a) an embryonal cell carcinoma of the right testis limited to the testis with lymphovascular invasion of pathological stage pT2 (Figure 1): staining with antibodies showed CD30(+) and a-FP(−); (b) a seminoma of the left testis, limited to the testis without invasion of the tunica albuginea or vascular invasion of pathological stage pT1 (Figure 2): staining with antibodies showed CD117(+), CD30(−), and a-FP(−). On both testicles intratubular germ cell neoplasia of unclassified type (IGCNU) was present. The patient was referred to an oncologist and was submitted to two cycles of adjuvant combined chemotherapy with bleomycin, etoposide, and cisplatin (BEP). His follow-up consisted of physical examination, serum markers, chest X-ray, and CT of the abdomen, according to the EAU Guidelines recommended follow-up schedule. Six months after the operation no residual tumor or recurrence was observed, neither local nor systematic. Finally the patient is under a strict endocrinologist follow-up for the management of his hypogonadism state.

## 3. Discussion

Testicular cancer is the most common solid malignancy in young men of 15–35 years old. It represents between 1% and 1.5% of male neoplasms and 5% of urological tumors in general, with 3–10 new cases occurring per 100,000 males/per year in Western society [1–3]. The mortality of testicular cancer has decreased significantly over the past years, mostly due to improved diagnostic methods and the use of adjuvant treatments [5]. The histological type varies, although there is a clear predominance (90–95%) of germ cell tumors [1]. The main risk factor for the development of a testicular cancer is cryptorchidism, with a relative risk of 3,5–17,1 [6, 7]. The incidence of bilateral testicular germ cell tumor (BGCT) is 1–4%, in selected series, as presented in Table 1 [6, 8–13], with 80–85% of them occurring metachronously and 10–15% occurring synchronously, accounting for less than 0,5% of all testicular cancers [7, 8, 14]. BGCT are reported to present in younger males and as documented by one report at a median age of 29 years compared to a median age of 34 years for solitary testis cancer [15]. The first documented case of bilateral testicular germ cell tumors was reported by Bidard in 1853 [16]. Since then, many series have been published, showing that synchronous bilateral testicular cancer is a quite rare situation [4]. One of the largest series was presented by Holzbeierlein et al., with only 10 males out of 3984 having synchronous BGCT [12]. Another large study was presented by Che et al. from MD Anderson Cancer Center, over a study period from 1978 to 1999. They reported 2,431 patients with testis cancer, 24 of them with BGCT and four patients having synchronous tumors [6]. Synchronous BGCT tend to be of the same histologic type, mostly seminoma in 80% of the cases [17], and there are only a few cases of a patient affected by synchronous testicular cancer of different histology, with one being metastatic [18]. Bach et al. reported in 1983 a large series of BGCT, with the predominant histology type being seminoma (48%), followed by nonseminomatous testis cancer (15%) and nongerm cell tumors (22%), and with different histology (15%) [19]. Although, synchronous BGCT were once thought to present at a higher stage disease in contrast to those with unilateral testicular cancer [4], this has not been demonstrated in multiple series reviewed [7] and this is also presented in our case report. Morales-Barrera et al. presented 151 patients with testis cancer. Eight of them had BGCT, with only one (0,7%) being synchronous, and they concluded that patients with bilateral testicular cancer have similar survival rate to that of patients with unilateral cancer [20]. The first report of synchronous BGCT with different histology was by Coleman and MAcKeown Matsushima et al. in 1954 [16].

The latest review of the literature about synchronous BGCT with different histology was presented by Coli et al. in 2003. They reported 43 cases with the precise histological subtype, as diagnosed by the authors of the original paper [16]. Since then, five cases have been reported. In 2005, Hasebe et al. [21] presented a case of typical seminoma of the right testis and embryonal carcinoma of the left testis with metastatic lymph nodes, treated with bilateral orchiectomy and retroperitoneal lymph node dissection after 4 courses of systematic chemotherapy BEP. In 2006, Shoji et al. [22] reported a case of seminoma, embryonal carcinoma, yolk sac tumor, and immature teratoma in the right testis and seminoma in the left testis, with metastatic lymph nodes. The patient was treated with bilateral orchiectomy, followed postoperatively by two courses of BEP therapy and two courses of EP (etoposide, cisplatinum) therapy. In 2008, Lopez et al. [23] presented a case of synchronous bilateral BGCT, treated with bilateral orchiectomy, followed postoperatively by two courses of BEP therapy. In 2010, Yanagihara et al. [24] reported a case of seminoma and immature teratoma in the right testis and seminoma in the left testis and Kai et al. [18] reported a metastatic case. Thus, to the author's knowledge by searching in PubMed's database, our case is the 49th case of synchronous BGCT with different histology presented until now. Concerning the treatment options for synchronous BGCT, bilateral radical inguinal orchiectomy with high ligation of the spermatic cord is the standard of care [7]. This procedure generates infertility, need for indefinite androgen replacement therapy, and many other adverse effects due to castration and psychological disorders [4]. Bilateral orchiectomy with androgen substitution has been described by Tekin et al. for 11 patients, with no record of recurrence or other side effects of testosterone replacement therapy [25]. An alternative treatment method is an organ preserving surgery, a partial orchiectomy, which should be performed only in selected cases with surgical rules respected. The first hemiorchiectomy was performed by Richie in 1984. The tumor volume has to be less than 30% of the testicular volume (European Association of Urology Guidelines) or smaller than 25 mm confined to the testis [4] or smaller than 2 cm in diameter according to other studies [23] and preoperative testosterone levels should be normal. In those cases of conservative treatment, the rate of associated TIN (Testicular Intraepithelial Neoplasia) is high (at least up to 82%) with a 5-year probability for recurrence of 9%. Thus all patients must be treated with adjuvant radiotherapy (16–20 Gy) at some point or otherwise a frequent postoperative surveillance has been suggested [23, 26]. Infertility will result after radiotherapy and there is a risk of long-term Leydig cell insufficiency, so radiotherapy may be delayed in fertile patients who wish to father children. The German Testicular Cancer Intergroup recommends that a partial orchiectomy should always be considered [26]. Klatte et al. reported a large series from the University of Magdeburg in Germany of 612 patients with testis cancer, with 17 patients with bilateral involvement and six with synchronous disease. The authors concluded that partial orchiectomy may be performed in selected cases [27]. Tomita et al. presented eight patients with synchronous bilateral seminoma and advocated an alternative treatment choice, with radical orchiectomy for the larger testis tumor with the contralateral testis spared followed by three courses of BEP chemotherapy [17]. Bokemeyer et al. also confirmed the therapeutic role of chemotherapy in the contralateral testis [28]. There are several works reporting the protective role of systemic chemotherapy against the development of a second testicular tumor [5], with Oliver et al. suggesting that one cycle of adjuvant carboplatin can achieve similar relapse-free survival rates to prophylactic radiotherapy in patients with clinical stage I seminoma [29]. The treatment of patients with synchronous BGCT is based upon the clinical stage and the histopathological type of the tumors is determined by the most malignant component [16] and should not be different from the standard management of unilateral testicular carcinoma [4]. There are many adjuvant modalities that can be used after bilateral orchiectomy for synchronous BGCT. In the case of synchronous bilateral stage I seminomatous germ cell tumors, which are the most common type, surveillance, adjuvant radiotherapy, or platinum-based chemotherapy is reasonable options following orchiectomy. The current standard of care is adjuvant radiotherapy, especially when there is rete testis invasion [7]. Radiotherapy includes doses of 20–25 Gy, directed to the retroperitoneal lymph nodes with excellent local control. There are several series reporting adjuvant radiotherapy in bilateral seminoma [6, 9–11], the larger by Géczi et al. [13]. Surveillance is an option for stage I seminoma, now accepted as category 1 evidence in the USA. Currently there is not any literature supporting surveillance in bilateral seminoma. Chemotherapy can be used to prevent recurrence of seminoma. It seems to have no inferiority to radiotherapy with regard to 5-year recurrence-free survival (94,7% versus 96,0%) and causes a reduction in the number of contralateral testis cancers compared to the radiotherapy arm, with HR 0.22 (*P* = 0.03) [29]. In stage IIA/IIB seminoma chemotherapy with three cycles of BEP is an alternative to radiotherapy, with similar therapeutic result. When a nonseminomatous tumor is present in the case of BGCT, it is usually treated with adjuvant chemotherapy (BEP). There are series demonstrating the need of retroperitoneal lymphadenectomy in these cases. Whenever the stage is >IIC, three of four cycles of BEP should be used. In our case, the patient presented with a seminoma of stage I of low risk on one testis and an embryonal carcinoma of high risk, stage I, on the other testis. So, we decided to treat the patient with bilateral orchiectomy followed by two cycles of adjuvant BEP.

## 4. Conclusions

Synchronous bilateral germ cell tumors of the testicles are a quite rare situation accounting for less than 0,5% of all testicular cancers, while the synchronous bilateral testicular cancer of different histology is even more rare. They present with the same histologic type, usually as seminoma, and there are only a few reported cases reported with different histology. The management is the same regardless of the fact that whether the histology is the same or different in the two testicles. They present in younger males with similar survival to unilateral testis cancer and they are a challenging situation for the urologist. The standard of care is bilateral orchiectomy followed by an adjuvant modality based upon the clinical stage and the histopathological type.

## Figures and Tables

**Table 1:** Incidence of BGCT in selected series.

Author	Total number of testicular germ cell tumors	BGCT (%)	Synchronous BGCT
Patel et al. [9]	795	19 (2.3)	4
Coogan et al. [10]	2088	21 (1.0)	5
Ondruš et al. [11]	960	27 (2.8)	3
Che et al. [6]	2431	24 (1.0)	4
Holzbeierlein et al. [12]	3984	58 (1.5)	10
Theodore et al. [8]	2383	45 (4.0)	14
Géczi et al. [13]	2386	72 (3.0)	19
